# Genome Size Evolution and Dynamics in *Iris*, with Special Focus on the Section *Oncocyclus*

**DOI:** 10.3390/plants9121687

**Published:** 2020-12-01

**Authors:** Nour Abdel Samad, Oriane Hidalgo, Elie Saliba, Sonja Siljak-Yakovlev, Kit Strange, Ilia J. Leitch, Magda Bou Dagher-Kharrat

**Affiliations:** 1 Laboratoire Biodiversité et Génomique Fonctionnelle, Faculté des Sciences, Campus Sciences et Technologies, Université Saint-Joseph, Mar Roukos, Mkalles, BP: 1514 Riad el Solh, Beirut 1107 2050, Lebanon; Nour_A.S@hotmail.com (N.A.S.); eliesaliba@hotmail.fr (E.S.); 2 Ecologie Systématique Evolution, Université Paris-Saclay, CNRS, AgroParisTech, 91400 Orsay, France; Sonia.yakovlev@u-psud.fr; 3 Royal Botanic Gardens, Kew, Richmond, Surrey TW9 3AB, UK; K.Strange@kew.org (K.S.); I.Leitch@kew.org (I.J.L.); 4 Institut Botànic de Barcelona (IBB, CSIC-Ajuntament de Barcelona), Passeig del Migdia s.n., 08038 Barcelona, Spain

**Keywords:** East Mediterranean, genome size, *Iris*, Lebanon, phylogeny, *Oncocyclus*, continental radiation, West and Central Asia

## Abstract

Insights into genome size dynamics and its evolutionary impact remain limited by the lack of data for many plant groups. One of these is the genus *Iris*, of which only 53 out of c. 260 species have available genome sizes. In this study, we estimated the C-values for 41 species and subspecies of *Iris* mainly from the Eastern Mediterranean region. We constructed a phylogenetic framework to shed light on the distribution of genome sizes across subgenera and sections of *Iris*. Finally, we tested evolutionary models to explore the mode and tempo of genome size evolution during the radiation of section *Oncocyclus*. *Iris* as a whole displayed a great variety of C-values; however, they were unequally distributed across the subgenera and sections, suggesting that lineage-specific patterns of genome size diversification have taken place within the genus. The evolutionary model that best fitted our data was the speciational model, as changes in genome size appeared to be mainly associated with speciation events. These results suggest that genome size dynamics may have contributed to the radiation of *Oncocyclus* irises. In addition, our phylogenetic analysis provided evidence that supports the segregation of the Lebanese population currently attributed to *Iris persica* as a distinct species.

## 1. Introduction

The royal irises (*Iris* L. subgenus *Iris*, section *Oncocyclus* (Siemssen) Baker) have experienced a remarkable radiation across the rocky hillsides, steppes, and deserts of the Middle East (incl. the Eastern Mediterranean and Western Asia regions), giving rise to c. 33 species [1]. These irises exhibit a highly distinctive morphology with a short stem, small falcate leaves arranged in a fan-shaped structure, and, in proportion to the rest of the plant, an oversized solitary flower of varied and complex colour patterns. Flowers of usually dark colour harvest solar energy, a floral heat tightly linked to the night-sheltering bee pollination system which occurs in most species [2,3,4,5]. Shelter mimicry is a rare strategy otherwise restricted to orchids in the Euro-Mediterranean region [4]. A few *Oncocyclus* species (such as *Iris paradoxa* Steven) are pollinated through sexual deception—an even more specialised strategy—which is, like shelter mimicry, exclusive to orchids and royal irises in the Euro-Mediterranean region. Within the *Oncocyclus* irises, it has been suggested that sexual deception evolved from shelter mimicry [4]. The pollinator-mediated selection of floral traits is considered a major factor driving diversification in this group, although the extent to which it does is still debated [5,6].

Species of section *Oncocyclus* are generally strict endemics, typically occurring in a small number of scattered, disjunct populations, whose geographical isolation is enhanced by their pollination strategy and myrmecochory seed dispersal. Morphological divergence between populations usually follows a cline reflecting local adaptation to environment conditions; furthermore, this largely overlaps divergence between species, making it difficult to identify discrete species boundaries in these irises [7]. The magnitude of the problem has been highlighted by a recent molecular phylogeny of royal irises, wherein none of the five species represented by two or three subspecific entities were recovered as monophyletic [8]. Given that many royal irises are now threatened in the wild [9,10], this makes the need to better document the biodiversity of this *Iris* section an urgent priority for optimising conservation strategies.

While the genus *Iris* is typically reported to exhibit considerable karyotype diversity (e.g., in chromosome numbers and frequency of polyploidy and dysploidy [11]), section *Oncocyclus* is distinctive as it appears karyotypically stable, with all species presenting a strikingly similar, bimodal, asymmetric chromosome complement of 2*n* = 20 [12]. Available cytogenetic data for *Oncocyclus* irises also support this view, as shown by a recent study [13], which highlighted the presence of three 35S rDNA loci in the royal irises, which is one more locus than reported for other *Iris* species from the Eastern Mediterranean region.

To our knowledge, there are currently only three genome size estimates reported for species belonging to section *Oncocyclus* [14]: *I. lortetii* Barbey ex Boiss. (2C = 15.46 pg), *I. sofarana* Foster (2C = 11.5 pg) and *I. sofarana* subsp. *kasruwana* (Dinsm.) Chaubhary, G.Kirkw. & C.Weymouth (2C = 16.36 pg). These values rank average to low compared with the C-value estimates for 39 *Iris* species (c. 15%) reported in the Plant DNA C-values database release 7.1. [15,16] which range from 2.1 to 56.4 pg/2C, with a mean of 18.11 pg/2C (calculated with the “prime estimate” search option that gives one value per species and cytotype). The growing pool of evidence aiming to link changes in genome size to speciation and speciation rate (see [17] for an overview) and to species radiation [18,19,20,21] makes a closer study of genome size dynamics within section *Oncocylus* worthwhile.

In this study, we have undertaken a survey of C-values across *Iris* species, with a special emphasis on subgenus *Iris* and particularly on section *Oncocyclus* which is placed within this subgenus. The aims of the study were to (i) assess the extent of genome size diversity in these taxa, and (ii) provide insights into genome size evolution in section *Oncocyclus*.

## 2. Results

### 2.1. Iris Phylogenetic Reconstruction

**Figure 1:**
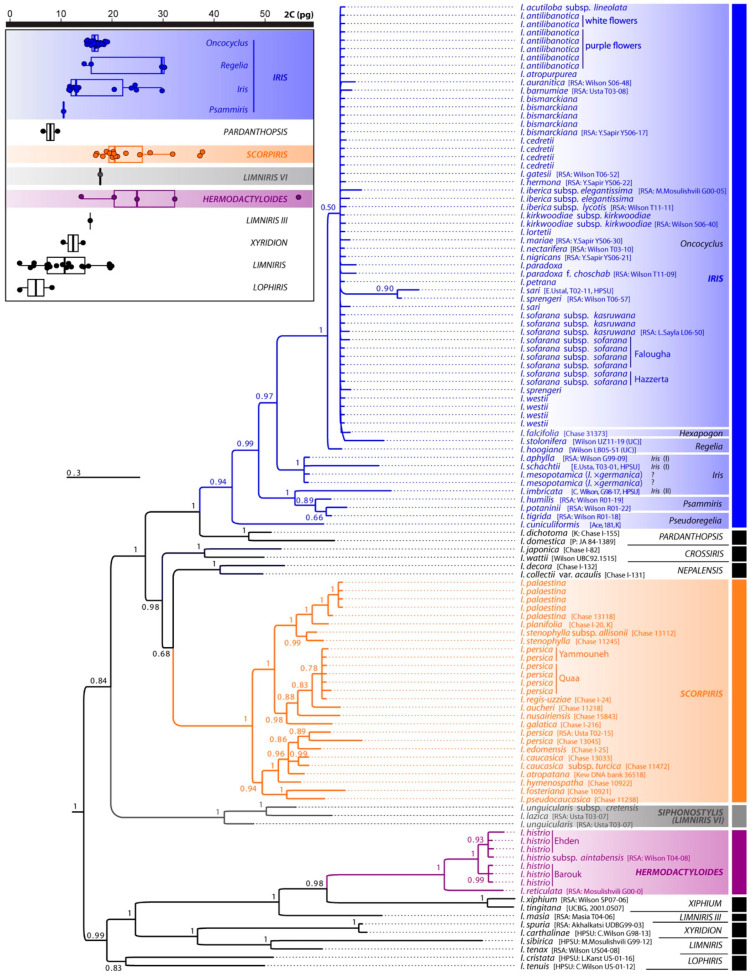
Majority-rule consensus phylogeny of post-burn trees of *Iris* resulting from the analysis of the *matK*-*trnK* and *trnL*-*trnF* combined dataset. Posterior probabilities > 0.50 are indicated on nodes. For sequences gathered from GenBank, the voucher reference is given in brackets after the species name. Infrageneric classification follows [24]. Upper left: boxplots with individual jitter values for genome size accessions, depicting the distribution of mean 2C-values per species (and cytotypes when genome size data suggest different ploidy levels) throughout the *Iris* subgenera and sections which have genome size data available (Table S2).

Nucleotide sequence data for 20 *Iris* species and subspecies have been deposited in the DDBJ/EMBL/GenBank (accessions MW110365–MW110415 for *trnL*-*trnF*, MW110416–MW110469 for *matK*-*trnK*; Table S1). The phylogenetic tree resulting from the Bayesian analysis of the combined dataset is presented in Figure 1. Separate analyses of the different markers (data not shown) did not reveal any incongruences with branch support. Our results are consistent with the subgeneric and sectional divisions of the genus *Iris*, although in subgenus *Iris* most accessions fell in an unresolved polytomy (Figure 1). The analysis of multiple accessions per species yielded no conflict, except for the Lebanese populations from Yammouneh and Quaa attributed to *Iris persica* L. These appeared more closely related to *I. regis-uzziae* Feinbrun, *I. aucheri* (Baker) Sealy, *I. nusairiensis* Mouterde and *I. galatica* Siehe than to the two Turkish accessions of *Iris persica* (accessions “Usta T02-15”, [22]; “Chase 13045”, [23]; Figure 1).

### 2.2. Genome Size Diversity across Iris Subgenera and Sections

**Figure 2:**
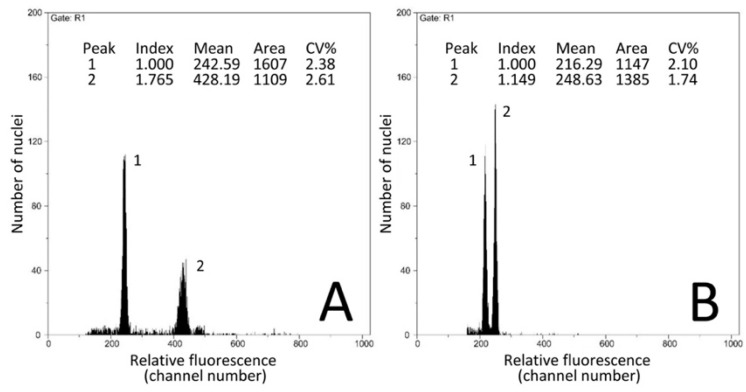
Examples of flow histograms obtained from estimating genome sizes in *Iris*. (**A**) Flow histogram obtained from analysing *I. korolkovii* (16.03 pg/2C, peak 2) using *Pisum sativum* (9.09 pg/2C, peak 1) as the calibration standard. (**B**) Flow histogram obtained from co-processing *I. acutiloba* subsp. *linolata* (16.73 pg/2C, peak 1) and *I. sprengeri* (19.20 pg/2C, peak 2).

We estimated C-values for 50 accessions of 41 *Iris* species and subspecies, mostly from the Eastern Mediterranean region, including 37 populations of taxa belonging to section *Oncocyclus* (27 species, three of them with two subspecies; Table 1). Since the genome size of *I. sofarana* Foster published in [14] appeared much smaller (2C = 11.5 pg) than any other *Iris* from section *Oncocyclus*, we reassessed this value measuring the exact same population of Dahr El-Baydar with the same calibration standard and extraction buffer, and obtained 2C = 16.22 pg. With this new value, the genome size in section *Oncocyclus* varied only 1.27-fold, ranging from 2C = 15.13 pg in *I. camillae* Grossh. to 2C = 19.24 pg in *I. bismarckiana* Damman & Sprenger. We confirmed the overall genome size variation of *Oncocyclus* irises by co-processing leaves from two individuals with genome sizes that cover nearly the full range of C-values estimated for this section (Figure 2).

In addition to the 50 populations whose genome sizes were assessed in this study, we gathered published data for 53 species ([14,33,34,35,36,37,38,39,40,41,42] from the Plant DNA C-values database release 7.1. [15], and more recently published estimates [43,44]; Table S2). We did not include two accessions of imprecise species identification, *I.* aff. *maracandica* (Vved.) Wendelbo and *I.* aff. *orchioides* Carrière, nor the *I. sofarana* accession from [14] that has been reassessed in the present study (see above). The distribution of genome sizes throughout *Iris* subgenera and sections is depicted in Figure 1.

### 2.3. Genome Size Evolution in Oncocyclus Section

**Figure 3:**
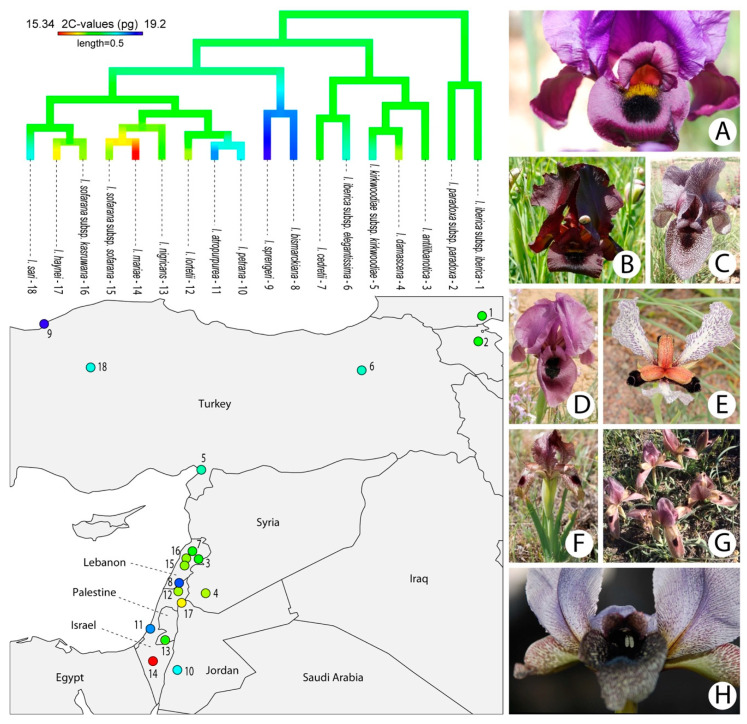
Reconstruction of ancestral genome sizes across the phylogeny of section *Oncocyclus* together with the geographical locations of the populations studied (**left**) and pictures illustrating the floral phenotype diversity amongst royal irises (**right**). Geographical locations of *I. iberica* subsp. *iberica* and *I. sari* accessions, of unknown origin, were taken from the literature. (**A**) *I. antilibanotica.* (**B**) *I. atropurpurea* (credit: Mat Knight and Zachi Evenor). (**C**) *I. cedretii.* (**D**) *I. mariae* (credit: חדוה שנדרוביץ). (**E**) *I. paradoxa* (credit: C.T. Johansson). (**F**) *I. sari* (credit: Zeynel Cebeci). (**G**) *I. sprengeri* (credit: Kohran1923). (**H**) *I. westii*.

Ancestral genome size reconstruction suggested that both increases and decreases in genome size have taken place during the evolutionary history of section *Oncocyclus* (Figure 3), indicating a certain degree of lability of this trait within the 1.27-fold range of variation encountered. Pagel’s λ estimate is close to zero (λ = 5.73 × 10^−5^) with a *p*-value of 1, suggesting that there is essentially no phylogenetic signal in genome size among *Oncocyclus* irises. Blomberg’s K estimate of <1 indicates that the genome sizes of species are less similar than expected under a random drift model (K = 0.43, *p* = 0.207). Taken together, these data indicate that the trends in genome size evolution within section *Oncocyclus* appear largely unrelated to phylogeny, and are also unlikely to be accounted for by random processes alone. The evolutionary model that best fitted our data was the speciational model (pure phylogenetic/equal model; Table 2), where changes in genome size appear to occur most frequently during speciation events.

## 3. Discussion

### 3.1. Iris Displays a Great Diversity of C-Values but They Are Unequally Distributed across Subgenera and Sections

This study increased the number of species with available genome size data from 53 to 89, raising the coverage from c. 20% up to c. 34% of species. Despite nearly doubling the number of species with C-value data, the overall range in C-values remained unchanged (2.1–56.4 pg/2C; i.e., 26.86-fold). Yet this study has highlighted, for the first time, the very unequal distribution of genome size diversity across the different *Iris* subgenera and sections which currently have genome size data (Figure 1). Such findings suggest that lineage-specific patterns of genome size diversification have taken place within *Iris*. Unfortunately, the available phylogenetic and genome size data are still too fragmentary to precisely characterise these patterns and draw any firm conclusion as to their impact on the evolution of *Iris* as a whole. Nevertheless, we have provided novel insights into this by focusing on section *Oncocyclus*, given the new genome size estimates provided here and the recent phylogenetic and cytogenetic data of [8,13].

Taken together, *Oncocyclus* irises are currently the most extensively studied of the *Iris* sections from a genome size perspective, with data for c. 82% of recognised species, yet they present an extremely narrow C-value range (1.27-fold, 27 out of c. 33 recognised species with genome size), less than half the range encountered in two closely related sections: *Regelia* (2.84-fold, 5 out of 8 recognised species with genome size) and *Iris* (2.68-fold, 13 out of c. 42 recognised species with genome size) from the same subgenus. There is currently only one genome size estimate for section *Psammiris*, and no genome size data for species belonging to the other two sections in subgenus *Iris* (i.e., *Pseudoregelia* and *Hexapogon*), and so insights into genome size diversity in these sections are currently unclear.

### 3.2. Genome Size Dynamics Could Have Contributed to the Radiation of Oncocyclus Irises

Previous studies have highlighted a very stable karyotype and ribosomal DNA loci number in section *Oncocyclus*, and postulated karyological and cytogenetic stasis in this group [12,13]. Consistent with this assumption, we uncovered a particularly narrow range of C-values in section *Oncocyclus* (from 15.13 to 19.24 pg/2C; Table 1). Yet even though the genome size variation is of modest magnitude, the ancestral genome size reconstruction analysis does suggest that there is a certain degree of lability in this trait (as indicated by changes in the colour of the branches in the phylogeny shown in Figure 3), with evidence of increases in 10 species and decreases in 8 species from an ancestral genome size of 17.29 pg/2C (Figure 3).

Furthermore, the fact that the speciational model of trait evolution was the best fit to our data (Table 2) indicates that genome size dynamics have likely participated in the radiation of section *Oncocyclus*. This may seem surprising at first, given the limited genome size range; however, recent studies have suggested that it is the rate of variation in genome size rather than the absolute genome size value that may play a role in lineage diversification, shifting the emphasis to the importance of genome size lability—or evolvability—as a potential driver contributing to plant evolution [45,46]. The fit of a speciational model for the evolution of a functional trait is usually interpreted in terms of the adaptive value for the trait in question (e.g., spur length [47]). Genome size influences plants in myriad ways, at the nuclear, cellular, whole plant, and community levels. It impacts their phenotypic and ecological spectrums, and hence potentially also affects their evolvability and resilience to environmental change (reviewed in [17]). Such a diversity of effects on traits potentially subject to selection makes it difficult to address the functional outcomes of changes in genome size (with the notable exception of the largest genomes [48]). It is only by gaining insights into different aspects of the radiation of the *Oncocyclus* irises (at the genetic, genomic, phenotypic, and ecological levels) and by integrating all this information that we will be able to improve our understanding of how genome size has contributed to the diversification of the group.

### 3.3. Lebanese Populations Attributed to Iris persica (Subgenus Scorpiris) Should Be Segregated into a Distinct Species

This study has brought the first molecular phylogenetic insights into the current debate on the taxonomic status of Lebanese populations previously assigned to *I. persica*. The phylogenetic data (Figure 1) show that the sample from Turkey falls into a separate, yet well-supported, clade from the Lebanese individuals analysed here, and hence clearly support the consideration of the Lebanese populations as taxonomically distinct from *I. persica*. This consideration was until now mainly based on differences in chromosome numbers between populations, with 2*n* = 24 reported for the Lebanese population of Quaa [13], while *I. persica* from Syria and Turkey presented 2*n* = 20 and 36, respectively [31,32]. We also found differences in genome size, with 18.89 and 19.05 pg/2C for the two Lebanese populations and 20.99 pg/2C for the Turkish one (Table 1).

It has yet to be determined whether these Lebanese irises correspond to a new species or if they belong to *I. wallisiae*, as hypothesised by some authors [31]. Unfortunately, *I. wallisiae* has not been sequenced so far, and its genome size has not been measured either; however, the difference in chromosome numbers observed between the Lebanese irises (2*n* = 24, [13]) and *I. wallisiae* (2*n* = 22, [31]) tends to suggest the existence of a new species.

In order to better understand the evolutionary history of these irises and to clarify their taxonomic status, it is necessary to carry out additional studies on an extended sampling and to consider the possibility of gene flow and hybridisation, which have been reported in other sections of *Iris* [49,50]. This is not possible with the data presented here, as our phylogeny is based exclusively on plastid markers.

## 4. Materials and Methods

### 4.1. Material

Plants studied were collected from natural populations in different regions of Lebanon. Additional accessions were obtained from the living collections and the DNA Bank held at the Royal Botanic Gardens, Kew (RBGK), and from the private collection of Frédéric Dépalle. Table 1 gives the provenance of the 50 *Iris* populations sampled for genome size, and Table S1 the provenance of the 23 *Iris* populations sampled for the molecular phylogenetic analyses.

### 4.2. Molecular Phylogenetic Reconstruction

DNA was isolated from 50–170 mg of frozen or fresh leaves using a modified cetyltrimethyl ammonium bromide (CTAB) method [51]. Plastid *trnL*-*trnF* and *matK*-*trnK* regions were amplified using the primers trnL-c (5′-CGAAATCGGTAGACGCTACG-3′) and trnL-f (5′-ATTTGAACTGGTGACACGAG-3’) [52], matK19F (5′-CGTTCTGACCATATTGCACTATG-3′) [53], and trnK-2R (5′-AACTAGTCGGATGGAGTAG-3′) [54]. PCR reactions were performed in a volume of 50 µL containing 10 µL of 5× Phire Reaction Buffer, 10 mM of each dNTP, 100 µM primers, 20–50 ng genomic DNA template, and 1 µL Phire Hot Start II DNA Polymerase (F122-S, Thermo Fisher Scientific Inc., Waltham, MA, USA). The PCR program had an initial strand separation step at 98 °C for 30 s, followed by 35 cycles of denaturation at 98 °C for 5 s, annealing at 57 °C for 5 s, and elongation at 72 °C for 15 s; the final step was at 72 °C for 1 min. Purified PCR products were sequenced by Eurofins MWG, France using the Sanger method on an ABI 3730xL platform.

The dataset, comprising the new sequences generated here together with sequences obtained from GenBank, was aligned using the default settings on the Guidance webserver [55] and manually adjusted with BioEdit software [56]. It comprises the *matK*-*trnK* region for 117 accessions (representing 1842 characters) and the *trnL*-*trnF* region for 94 accessions (1028 characters). *Dietes robinsoniana* (F.Muell.) Klatt was included in the analysis as an outgroup species. Bayesian inference (BI) was carried out using MrBayes version 3.1.2 [57] on the CIPRES server [58] with partitions by region (*trnL*-*trnF*, *matK*, and *trnK*) and, when applicable, by codon position. The best-fit model of nucleotide substitutions selected with jModelTest 0.1 [59,60] using the Akaike Information Criterion (AIC) was GTR + I + G for all region partitions. For each analysis, four Markov chains were run simultaneously for 30 × 10^6^ generations, and these were sampled every 700 generations. Analyses were checked for convergence in Tracer v.1.6 [61]. Data from the first 10,000 generations were discarded as the burn-in period in each analysis, and the remaining pooled samples were used to build the 50% majority rule consensus trees and to calculate the posterior probability (PP) of nodes.

### 4.3. DNA Content Assessment

Genome size was measured using the one-step flow cytometry procedure [62] with modifications as described in [14], or, for RBGK accessions, as described in [63], with a Partec Cyflow SL3 flow cytometer (Partec GmbH, Münster, Germany). For each species studied, Table 1 gives information on the specific plant species used as the calibration standard and the specific extraction buffer. Only estimates where the peak quality, expressed as a coefficient of variation (CV%), was less than 5% were considered acceptable.

### 4.4. Tempo and Mode of Character Evolution

Box-plots showing the distribution of genome size values in the nine subgenera of *Iris* and four sections within subgenus *Iris* were generated with the package ggplot2 [64] implemented in R v.3.2.2 [65] using the new genome size assessments together with previously published data (Table S2).

Analyses focused on section *Oncocyclus* were conducted using the phylogenetic inference of [8], pruned with BayesTrees v.1.3 [66] to the set of species with available genome sizes, and made ultrametric using the chronos function in the package ape [67] implemented in R v.3.2.2 [65]. Subspecies of *I. iberica* Steven and *I. sofarana* were kept in the pruned tree since they do not group with their conspecifics in the phylogeny of [8]. Ancestral character states were reconstructed with the Phytools package of R [68] implemented in R v.3.2.2 [65] under ML using the fastAnc and contMap commands. The strength of the phylogenetic signal for genome size was estimated using Pagel’s λ and Blomberg’s K (phylosig function of Phytools). The Pagel’s λ method is used to transform the original phylogeny (using the λ parameter) so that it best predicts the distribution of a given trait on the phylogeny under a Brownian Motion model of trait evolution [69]. The null hypothesis is λ = 0, indicating no phylogenetic signal. Blomberg’s K method quantifies the degree to which variation in a trait is predicted by the structure of a given phylogeny under a Brownian Motion model of trait evolution [69]. The continuous-character model evaluation and testing (CoMET) module [70] implemented in MESQUITE V. 3.61 [71] was used with default parameters to determine which model of evolution best fitted our data.

## 5. Conclusions

This study provides new insights into genome size evolution in *Iris* at different taxonomic levels (i.e., genus and section), and advocates for continuing the effort to complete the coverage of C-value data within the genus. As our understanding of genome size dynamics grows, it is increasingly clear that genome size is an essential trait that should be taken into account in the study of evolutionary processes [17,21,72].

## Figures and Tables

**Table 1:** Genome size data for the 50 *Iris* accessions studied in the current work. Where available, chromosome counts and ecological data are given.

*Iris* (Subgenus, Section, Species)		2C in pg (SD) ^1^	St ^2^	Bu ^3^	2*n* ^4^	P ^5^	Origin
**Subgenus *Iris***							
	**Section *Oncocyclus***						
	*I. acutiloba* subsp. *acutiloba*	16.72 (0.17)	2	2	20	1	Azerbaijan: Jeyrankechmaz, Leg. F. Dépalle
	*I. acutiloba* subsp. *lineolata*	16.73 (0.10)	1	1	20	1	Armenia (RBGK 2012-1109)
	*I. acutiloba* subsp. *lineolata*	15.94 *	2	2	20	1	Armenia: Gandja, Leg. F. Dépalle
	*I. antilibanotica*	16.83 (0.54)	3	2	20 *	1	Lebanon: Kheibeh-Baalbeck, 1337 m
	*I. antilibanotica*	17.05 (0.54)	3	2	20 *	1	Lebanon: Kheibeh-Baalbeck, 1337 m
	*I. assadiana*	17.44 (0.56)	3	2	20	1	Syria: Sadad, Leg. F. Dépalle
	*I. atropurpurea*	18.53 (0.14)	1	1	20	1	Israel (RBGK 1998-2808)
	*I. barnumiae* x *I. paradoxa f. choschab*	17.31(0.05)	1	1	20	1	Unknown Leg. Ray Drew (RBGK s.n.)
	*I. bismarckiana*	18.24 (0.59)	3	2	20 *	1	Lebanon: Sarada, 435 m
	*I. bismarckiana*	19.24 (0.54)	2	2	20	1	Jordania: Ajloun, Leg. F. Dépalle
	*I. camillae*	15.13 (0.49)	3	2	20	1	Azerbaijan: Tovuz, Leg. F. Dépalle
	*I. cedretii*	16.83 (0.5)	3	2	20 *	1	Lebanon: Bcharre, 1900 m
	*I. damascene*	16.44 (0.04)	3	2	20	1	Syria: Damas, Leg. F. Dépalle
	*I. haynei* var. *jordana*	16.20 (0.52)	3	2	20	1	Jordania: Umm Qais, Leg. F. Dépalle
	*I. iberica* subsp. *elegantissima*	17.89 (0.18)	1	1	20	1	Turkey: 2200 m (RBGK 1999-4347)
	*I. iberica* subsp. *iberica*	17.25 (0.05)	1	1	20	1	Unknown (RBGK 2002-2632)
	*I. kirkwoodiae* subsp. *kirkwoodiae*	17.83 (0.06)	1	1	20	1	Turkey (RBGK 1994-2407)
	*I. kirkwoodiae*	17.25 (0.15)	2	2	20	1	Syria: St. Simeon, Leg. F. Dépalle
	*I. lortetii*	16.53 (0.19)	3	2	20 *	1	Lebanon: Mays el Jabal, 640 m
	*I. lortetii*	16.38 (0.29)	2,3	2	20	1	Israel: Ayelet-Hashahar, Leg. F. Dépalle
	*I. mariae*	15.34 (0.5)	3	2	20	1	Israel: Sede Boker, Leg. F. Dépalle
	*I. meda*	15.21 (0.49)	3	2	20	1	Iran: Baqloujeh Sardar, Leg. F. Dépalle
	*I. mirabilis*	15.69 (0.10)	2	2	20	2	Iran: Ayerandibi, Leg. F. Dépalle
	*I. nigricans*	16.68 *	3	2	20	1	Palestine: Bani Naim, Leg. Dr. Khalid Sawalha
	*I. paradoxa*	17.32 (0.08)	1	1	20	2	Armenia (RBGK 1977-4470)
	*I. petrana*	18.17 (0.11)	1	1	20	1	Unknown (RBGK 1990-3180)
	*I. petrana*	18.01 (0.48)	2	2	20	1	Jordania: Shawbak, Leg. F. Dépalle
	*I. samariae*	16.56 (0.04)	3	2	20	1	Israel: Majdal, Leg. F. Dépalle
	*I. sari*	18.03 (0.11)	1	1	20	1	Unknown (RBGK 2011-1955)
	*I. schelkovnikowii*	16.11 (0.04)	2	2	-	1	Azerbaijan: Mingachevir, Leg. F. Dépalle
	*I. sofarana* subsp. *kasruwana*	16.68 (0.53)	3	2	20 *	1	Lebanon: Ehmej, 1217 m
	*I. sofarana* subsp. *sofarana*	16.22 (0.37)	3	2	20 *	1	Lebanon: Dahr El-Baydar, 1640 m
	*I. sofarana* subsp. *sofarana*	16.90 (0.49)	3	2	20 *	1	Lebanon: Hazzerta, 1530 m
	*I. sprengeri*	19.20 (0.16)	1	1	-	1	Turkey: 1000 m (RBGK 2011-1958)
	*I. westii*	16.00 (0.75)	3	2	20 *	1	Lebanon: Tawmet Jezzine, 1300 m
	*I. yeruchamensis*	16.54 (0.05)	3	2	-	1	Israel: Yerocham, Leg. F. Dépalle
	*I. yebrudii* subsp. *yebrudii*	16.20 (0.11)	3	2	20	1	Syria: Yebrud, Leg. F. Dépalle
	**Section *Regelia***						
	*I. afghanica*	14.67 (0.47)	3	2	22	3	Afghanistan, Leg. F. Dépalle
	*I. hoogiana*	30.31 (0.13)	1	1	-	3	Tajikistan (RBGK 2010-2098)
	*I. korolkovii*	16.03 (0.03)	4	1	-	3	Unknown (RBGK 2010-2101)
	*I. lineata*	29.77 (0.04)	1	1	-	3	Tajikistan (RBGK 1993-3285)
	*I. stolonifera*	30.24 (0.11)	1	1	-	3	Pamir (RBGK 1984-91)
	**Section *Iris***						
	*I. albicans*	24.84 (0.65)	3	2	44–48	3	Lebanon: Aley, 880 m
	*I. scariosa*	13.09 (0.18)	1	1	-	3	Kazakhstan (RBGK 2014-643)
	**Section *Psammiris***						
	*I. bloudovii*	10.66 (0.13)	1	1	16, 26	3	Siberia (RBGK 2004-2257)
**Subgenus *Limniris***							
	*I. longipetala*	15.84 (0.19)	2	2	-	3	Iran: Jazvanaq, Leg. F. Dépalle
**Subgenus *Scorpiris***							
	*I. persica*	20.99 (0.05)	1	1	20, 36	3	Turkey: 1200 m (RBGK 2014-1875)
	*I. persica*	18.89 (0.32)	3	2	24 *	3	Lebanon: Quaa, 700 m
	*I. persica*	19.05 *	3	2	-	3	Lebanon: Yammouneh
	*I. regis-uzziae*	22.80 (0.03)	1	1	20	3	Israel: 500 m (RBGK 1987-2212)

^1^ Genome size: asterisk indicates when a single measurement has been done. ^2^ Calibration standard: (1) *Allium cepa* 2C = 34.89 pg [25], (2) *Artemisia arborescens* 2C = 11.43 pg [26], (3) *Triticum aestivum* 2C = 30.90 pg [27], (4) *Pisum sativum ‘*Ctirad’ 2C = 9.09 pg [25]. ^3^ Buffer: (1) GPB [28] with 3% PVP-40 and 8% Triton added, (2) Galbraith’s buffer [29]. ^4^ Chromosome number: asterisk indicates data determined in [13] on the same individuals measured for genome size; other data are from the Chromosome counts database [30], and, for *I. persica*, from [31,32]. ^5^ Pollination strategy: (1) shelter mimicry, (2) sexual deception, (3) other pollination strategy, from [4].

**Table 2:** Evaluation of evolutionary models for genome size in section *Oncocyclus* by the CoMET module. The best-fitting model (lowest Akaike Information Criterion; AIC) is indicated in bold.

Model		
**Phylogenetic Signal**	**Tempo of Trait Change**	**AIC**
	Distance	27.922
Non phylogenetic	Equal	22.243
	Free	165.507
	Distance	25.527
**Pure phylogenetic**	**Equal**	**19.454**
	Free	165.702
	Distance	110.326
Punctuated	Equal	32.232
	Free	142.326

## Supplementary Materials

The following are available online at https://www.mdpi.com/2223-7747/9/12/1687/s1, Table S1, GenBank accession numbers of the *Iris* sequenced in this study. Table S2, compendium of genome size data (2C-values) currently available for *Iris*, together with information on the subgeneric and sectional assignments of each species.
