# Supplementary material for: Genome Size Evolution and Dynamics in Iris, with Special Focus on the Section Oncocyclus
Source: Plants (Basel). 2020 Dec 1;9(12):1687. doi: 10.3390/plants9121687 (PMC7760388; doi:10.3390/plants9121687)
Supplement: Supplementary file 1 [file plants-09-01687-s001.pdf]

Table S1. GenBank accession numbers of the *Iris* sequenced in this study.

Taxa	Population [individual]	*trnL-trnF*	*matK-trnK*
Iris acutiloba subsp. *lineolata*	ARMENIA (RBGK 2012-1109)	MW110370	MW110422
Iris antilibanotica	LEBANON: Kheibeh-Baalbeck, 1337 m, white flowers [ind1]	MW110371	MW110423
Iris antilibanotica	LEBANON: Kheibeh-Baalbeck, 1337 m, white flowers [ind2]	MW110372	MW110424
Iris antilibanotica	LEBANON: Kheibeh-Baalbeck, 1337 m, purple flowers [ind1]	MW110373	MW110425
Iris antilibanotica	LEBANON: Kheibeh-Baalbeck, 1337 m, purple flowers [ind2]	MW110374	MW110426
Iris antilibanotica	LEBANON: Kheibeh-Baalbeck, 1337 m, purple flowers [ind3]	MW110375	MW110427
Iris antilibanotica	LEBANON: Kheibeh-Baalbeck, 1337 m, purple flowers [ind4]	MW110376	MW110428
Iris antilibanotica	LEBANON: Kheibeh-Baalbeck, 1337 m, purple flowers [ind5]	MW110377	MW110429
Iris atropurpurea	ISRAEL (RBGK 1998-2808)	MW110378	MW110430
Iris bismarkiana	LEBANON: Sarada, 435 m [ind1]	MW110379	MW110431
Iris bismarkiana	LEBANON: Sarada, 435 m [ind2]	MW110380	MW110432
Iris bismarkiana	LEBANON: Sarada, 435 m [ind3]	MW110381	MW110433
Iris bismarkiana	LEBANON: Sarada, 435 m [ind4]	MW110382	MW110434
Iris cedretii	LEBANON: Bcharre, 1900 m [ind1]		MW110435
Iris cedretii	LEBANON: Bcharre, 1900 m [ind2]	MW110383	MW110436
Iris cedretii	LEBANON: Bcharre, 1900 m [ind3]	MW110384	MW110437
Iris cedretii	LEBANON: Bcharre, 1900 m [ind4]	MW110385	MW110438
Iris histrio	LEBANON: Ehden [ind1]	MW110365	MW110416
Iris histrio	LEBANON: Ehden [ind2]	MW110366	MW110417
Iris histrio	LEBANON: Ehden [ind3]		MW110418
Iris histrio	LEBANON: Barouk [ind1]	MW110367	MW110419
Iris histrio	LEBANON: Barouk [ind2]	MW110368	MW110420
Iris histrio	LEBANON: Barouk [ind3]	MW110369	MW110421
Iris iberica subsp. *elegantissima*	TURKEY: 2200 m (RBGK 1999-4347)	MW110386	MW110439
Iris kirkwoodiae subsp. *kirkwoodiae*	TURKEY (RBGK 1994-2407)	MW110387	MW110440
Iris lortetii	LEBANON: Mays el Jabal, 640 m	MW110388	MW110441
Iris mesopotamica (*I.* ×*germanica*)	LEBANON: Mrouje, 1200 m [ind1]		MW110458
Iris mesopotamica (*I.* ×*germanica*)	LEBANON: Mrouje, 1200 m [ind2]	MW110405	MW110459
Iris palaestina	LEBANON: Joun, 460 m [ind1]	MW110407	MW110461
Iris palaestina	LEBANON: Joun, 460 m [ind2]	MW110408	MW110462
Iris palaestina	LEBANON: Joun, 460 m [ind3]	MW110409	MW110463
Iris palaestina	LEBANON: Joun, 460 m [ind4]	MW110410	MW110464
Iris paradoxa	ARMENIA (RBGK 1977-4470)	MW110389	MW110442
Iris persica	LEBANON: Yammouneh [ind1]		MW110465
Iris persica	LEBANON: Yammouneh [ind2]	MW110411	MW110466
Iris persica	LEBANON: Quaa, 700 m [ind1]	MW110412	MW110467
Iris persica	LEBANON: Quaa, 700 m [ind2]	MW110413	MW110468
Iris persica	LEBANON: Quaa, 700 m [ind3]	MW110414	MW110469
Iris persica	LEBANON: Quaa, 700 m [ind4]	MW110415	
Iris petrana	(RBGK 1990-3180)	MW110390	MW110443
Iris sari	(RBGK 2011-1955)	MW110391	MW110444
Iris sofarana subsp. *kasruwana*	LEBANON: Ehmej, 1217 m [ind1]	MW110392	MW110445
Iris sofarana subsp. *kasruwana*	LEBANON: Ehmej, 1217 m [ind2]	MW110393	MW110446
Iris sofarana subsp. *sofarana*	LEBANON: Dahr El Baydar, 1640 m [ind1]	MW110394	MW110447
Iris sofarana subsp. *sofarana*	LEBANON: Dahr El Baydar, 1640 m [ind2]	MW110395	MW110448
Iris sofarana subsp. *sofarana*	LEBANON: Dahr El Baydar, 1640 m [ind3]	MW110396	MW110449
Iris sofarana subsp. *sofarana*	LEBANON: Dahr El Baydar, 1640 m [ind4]	MW110397	MW110450
Iris sofarana subsp. *sofarana*	LEBANON: Hazzerta, 1530 m [ind1]	MW110398	MW110451
Iris sofarana subsp. *sofarana*	LEBANON: Hazzerta, 1530 m [ind2]	MW110399	MW110452
Iris sprengeri	TURKEY: 1000 m (RBGK 2011-1958)	MW110400	MW110453
Iris unguicularis subsp. *cretensis*	LEBANON: Baadarane, 1100 m	MW110406	MW110460
Iris westii	LEBANON: Tawmet Jezzine, 1300 m [ind1]	MW110401	MW110454
Iris westii	LEBANON: Tawmet Jezzine, 1300 m [ind2]	MW110402	MW110455

Iris westii	LEBANON: Tawmet Jezzine, 1300 m [ind3]	MW110403	MW110456
Iris westii	LEBANON: Tawmet Jezzine, 1300 m [ind4]	MW110404	MW110457

Table S2. Compendium of genome size data (2C-values) currently available for *Iris*, together with information on the subgeneric and sectional assignments of each species. The infrageneric classification follows Wilson (2011). Published genome size values were obtained from the Plant DNA C-values database release 7.1 using the "all estimates" search option (Leitch et al. 2019), Choi et al. (2020) and Weber el al. (2020). We removed two accessions of imprecise species identification, *I.* aff. maracandica and *I.* aff. *orchioides*, and the *Iris sofarana* accession from Bou Dagher-Karrat et al. (2013) that has been reassessed in the present study. The final dataset includes new and published genome sizes for 89 *Iris* species.

Taxon	Subgenus	Section	2C (pg)	Reference for genome size data
Iris acutiloba subsp. *acutiloba*	*Iris*	*Oncocyclus*	16.72	This study
Iris acutiloba subsp. *lineolata*	*Iris*	*Oncocyclus*	15.94	This study
Iris acutiloba subsp. *lineolata*	*Iris*	*Oncocyclus*	16.73	This study
Iris adriaca	*Iris*	*Iris*	12.64	Weber et al. 2020
Iris afghanica	*Iris*	*Regelia*	14.67	This study
Iris albicans	*Iris*	*Iris*	24.84	This study
Iris albomarginata	*Scorpiris*		20.3	Hanson et al. 1999, unpublished
Iris albomarginata	*Scorpiris*		37.7	Hanson et al. 1999, unpublished
iris antilibanotica	*Iris*	*Oncocyclus*	16.83	This study
Iris antilibanotica	*Iris*	*Oncocyclus*	17.05	This study
Iris aphylla subsp. *hungarica*	*Iris*	*Iris*	20.53	Veselý et al. 2012
Iris assadiana	*Iris*	*Oncocyclus*	17.44	This study
Iris atropurpurea	*Iris*	*Oncocyclus*	18.53	This study
Iris barnumiae x I. paradoxa f. choschab	*Iris*	*Oncocyclus*	17.31	This study
Iris bismarckiana	*Iris*	*Oncocyclus*	18.24	This study
Iris bismarckiana	*Iris*	*Oncocyclus*	19.24	This study
Iris bloudovii	*Iris*	*Psammiris*	10.66	This study
Iris bosniaca	*Iris*	*Iris*	11.66	Siljak-Yakovlev et al. 2010
Iris bosniaca	*Iris*	*Iris*	12.04	Pustahija et al. 2013
Iris brevicaulis	*Limniris I (core Limniris)*		19.75	Kentner et al. 2003
Iris bucharica	*Scorpiris*		16.9	Hanson et al. 1999, unpublished
Iris bucharica	*Scorpiris*		17.3	Hanson et al. 1999, unpublished
Iris camillae	*Iris*	*Oncocyclus*	15.13	This study
Iris capnoides	*Scorpiris*		19	Hanson et al. 1999, unpublished
Iris cedretii	*Iris*	*Oncocyclus*	16.83	This study
Iris damascena	*Iris*	*Oncocyclus*	16.44	This study
Iris danfordiae	*Hermodactyloides*		32.3	Zonneveld et al. 2005
Iris dichotoma	*Pardanthopsis*		6.68	Choi et al. 2020
Iris domestica	*Pardanthopsis*		9.5	Choi et al. 2020
Iris ensata	*Limniris I (core Limniris)*		15.52	Choi et al. 2020
Iris foetidissima	*Limniris II (Xyridion)*		14.46	Veselý et al. 2012
Iris fulva	*Limniris I (core Limniris)*		19.57	Kentner et al. 2003
Iris germanica	*Iris*	*Iris*	11.74	Siljak-Yakovlev et al. 2010
Iris germanica	*Iris*	*Iris*	24.25	Weber et al. 2020
Iris germanica	*Iris*	*Iris*	24.9	Zonneveld et al. 2005
Iris graeberiana cv. White Fall	*Scorpiris*		27.5	Hanson et al. 1999
Iris graeberiana cv. Yellow Fall	*Scorpiris*		25.5	Hanson et al. 1999
Iris graminea	*Limniris II (Xyridion)*		10.58	Siljak-Yakovlev et al. 2010

Iris haynei var. *jordana*	*Iris*	*Oncocyclus*	16.2	This study
Iris hexagona	*Limniris I (core Limniris)*		19.59	Kentner et al. 2003
Iris histrio	*Hermodactyloides*		24.96	Bou Dagher-Kharrat et al. 2013
Iris histrio	*Hermodactyloides*		56.4	Goldblatt et al. 1984
Iris hoogiana	*Iris*	*Regelia*	30.31	This study
Iris iberica subsp. *elegantissima*	*Iris*	*Oncocyclus*	17.89	This study
Iris iberica subsp. *iberica*	*Iris*	*Oncocyclus*	17.25	This study
Iris illyrica	*Iris*	*Iris*	12.31	Siljak-Yakovlev et al. 2010
Iris kirkwoodiae	*Iris*	*Oncocyclus*	17.25	This study
Iris kirkwoodiae subsp. *kirkwoodiae*	*Iris*	*Oncocyclus*	17.83	This study
Iris koreana	*Limniris I (core Limniris)*		14.58	Choi et al. 2020
Iris korolkovii	*Iris*	*Regelia*	16.03	This study
Iris lacustris	*Limniris IV (Lophiris)*		8.4	Zonneveld et al. 2005
Iris laevigata	*Limniris I (core Limniris)*		14.24	Choi et al. 2020
Iris lineata	*Iris*	*Regelia*	29.77	This study
Iris longipetala	*Limniris III*		15.84	This study
Iris lortetii	*Iris*	*Oncocyclus*	15.46	Bou Dagher-Kharrat et al. 2013
Iris lortetii	*Iris*	*Oncocyclus*	16.38	This study
Iris lortetii	*Iris*	*Oncocyclus*	16.53	This study
Iris magnifica	*Scorpiris*		18.3	Hanson et al. 1999
Iris mariae	*Iris*	*Oncocyclus*	15.34	This study
Iris meda	*Iris*	*Oncocyclus*	15.21	This study
Iris mesopotamica	*Iris*	*Iris*	29.84	Bou Dagher-Kharrat et al. 2013
Iris minutoaurea	*Limniris I (core Limniris)*		7.7	Choi et al. 2020
Iris mirabilis	*Iris*	*Oncocyclus*	15.69	This study
Iris nelsonii	*Limniris I (core Limniris)*		20.04	Kentner et al. 2003
Iris nigricans	*Iris*	*Oncocyclus*	16.68	This study
Iris odaesanensis	*Limniris I (core Limniris)*		7.44	Choi et al. 2020
Iris orchioides	*Scorpiris*		19.38	Hanson et al. 1999
Iris orchioides	*Scorpiris*		20.95	Hanson et al. 1999
Iris oxypetala	*Limniris I (core Limniris)*		12.16	Choi et al. 2020
Iris palaestina	*Scorpiris*		20.72	Veselý et al. 2012
Iris palaestina	*Scorpiris*		21.56	Bou Dagher-Kharrat et al. 2013
Iris paradoxa	*Iris*	*Oncocyclus*	17.32	This study
Iris persica	*Scorpiris*		18.89	This study
Iris persica	*Scorpiris*		19.05	This study
Iris persica	*Scorpiris*		20.99	This study
Iris petrana	*Iris*	*Oncocyclus*	18.01	This study
Iris petrana	*Iris*	*Oncocyclus*	18.17	This study
Iris pseudacorus	*Limniris I (core Limniris)*		11.04	Bou Dagher-Kharrat et al. 2013
Iris pseudacorus	*Limniris I (core Limniris)*		11.2	Zonneveld et al. 2005
Iris pseudacorus	*Limniris I (core Limniris)*		11.33	Siljak-Yakovlev et al. 2010
Iris pseudacorus	*Limniris I (core Limniris)*		11.78	Choi et al. 2020
Iris pseudacorus	*Limniris I (core Limniris)*		12.8	Kenton and Owens 1988, unpublished
Iris pseudopallida	*Iris*	*Iris*	12.75	Siljak-Yakovlev et al. 2010
Iris pumila	*Iris*	*Iris*	13.62	Siljak-Yakovlev et al. 2010
Iris pumila	*Iris*	*Iris*	21.27	Veselý et al. 2012
Iris pumila	*Iris*	*Iris*	26.4	Zonneveld et al. 2005
Iris regis-uzziae	*Scorpiris*		22.8	This study
Iris reichenbachii	*Iris*	*Iris*	11.91	Siljak-Yakovlev et al. 2010
Iris reticulata	*Hermodactyloides*		14.1	Zonneveld et al. 2005

Iris reticulata cv. Harmony	*Hermodactyloides*		20.5	Zonneveld et al. 2005
Iris rossi	*Limniris I (core Limniris)*		7.46	Choi et al. 2020
Iris rubromarginata	*Iris*	*Iris*	13.21	Pustahija et al. 2013
Iris rubromarginata	*Iris*	*Iris*	13.21	Siljak-Yakovlev et al. 2010
Iris ruthenica	*Limniris I (core Limniris)*		4.84	Choi et al. 2020
Iris samariae	*Iris*	*Oncocyclus*	16.56	This study
Iris sanguinea	*Limniris I (core Limniris)*		10.46	Choi et al. 2020
Iris sari	*Iris*	*Oncocyclus*	18.03	This study
Iris scariosa	*Iris*	*Iris*	13.09	This study
Iris schelkovnikovii	*Iris*	*Oncocyclus*	16.11	This study
Iris setosa	*Limniris I (core Limniris)*		11.26	Choi et al. 2020
Iris sibirica	*Limniris I (core Limniris)*		4.2	Olszewska and Osiecka 1982
Iris sibirica	*Limniris I (core Limniris)*		8.95	Veselý et al. 2012
Iris sibirica	*Limniris I (core Limniris)*		9.61	Šmarda et al. 2014
Iris sintenisii	*Limniris I (core Limniris)*		8	Zonneveld et al. 2005
Iris sofarana subsp. *kasruwana*	*Iris*	*Oncocyclus*	16.36	Bou Dagher-Kharrat et al. 2013
Iris sofarana subsp. *kasruwana*	*Iris*	*Oncocyclus*	16.68	This study
Iris sofarana subsp. *sofarana*	*Iris*	*Oncocyclus*	16.22	This study
Iris sofarana subsp. *sofarana*	*Iris*	*Oncocyclus*	16.9	This study
Iris sprengeri	*Iris*	*Oncocyclus*	19.2	This study
Iris stenophylla	*Scorpiris*		17.06	Hanson et al. 2002, unpublished
Iris stenophylla	*Scorpiris*		21.93	Hanson et al. 2002, unpublished
Iris stenophylla	*Scorpiris*		23.35	Hanson et al. 2002, unpublished
Iris stolonifera	*Iris*	*Regelia*	30.24	This study
Iris tectorum	*Limniris IV (Lophiris)*		2.13	Zhang et al. 2013
Iris tubergeniana	*Scorpiris*		18.77	Hanson et al. 1999, unpublished
Iris tubergeniana	*Scorpiris*		22.1	Hanson et al. 1999, unpublished
Iris unguicularis subsp. *cretensis*	*Limniris VI (Siphonostylis)*		17.8	Bou Dagher-Kharrat et al. 2013
Iris uniflora	*Limniris I (core Limniris)*		4.92	Choi et al. 2020
Iris variegata	*Iris*	*Iris*	11.11	Veselý et al. 2012
Iris variegata	*Iris*	*Iris*	12.24	Siljak-Yakovlev et al. 2010
Iris versicolor	*Limniris I (core Limniris)*		2.1	Bai et al. 2012
Iris vicaria	*Scorpiris*		16.93	Hanson et al. 1999, unpublished
Iris vicaria	*Scorpiris*		30	Hanson et al. 1999, unpublished
Iris vicaria	*Scorpiris*		33.8	Hanson et al. 1999, unpublished
Iris westii	*Iris*	*Oncocyclus*	16	This study
Iris yebrudii subsp. *yebrudii*	*Iris*	*Oncocyclus*	16.2	This study
Iris yeruchamensis	*Iris*	*Oncocyclus*	16.54	This study
Iris zenaidae	*Scorpiris*		37.2	Hanson et al. 1999, unpublished
